# Nrf2在*EGFR*基因突变肺腺癌中的表达及其与EGFR-TKIs疗效间的相关性研究

**DOI:** 10.3779/j.issn.1009-3419.2014.02.15

**Published:** 2014-02-20

**Authors:** 翔 朱, 莉 梁, 晨 柳, 文琤 尹, 森 陈, 宝山 曹

**Affiliations:** 1 100191 北京，北京大学医学部病理学系，北京大学第三医院病理科 Department of Pathology, Peking University Health Science Center, Third Hospital, Beijing 100191, China; 2 100191 北京，北京大学第三医院病理科肿瘤化疗与放射病科 Department of oncology and radiation sickness, Cancer Center; Peking University Third Hospital, Beijing 100191, China; 3 100191 北京，北京大学第三医院放射科 Department of Radiology, Peking University Third Hospital, Beijing 100191, China

**Keywords:** 肺腺癌, EGFR, EGFR-TKIs, Nrf2, 生存期, Lung neoplasms, EGFR, EGFR-TKIs, Nrf2, Survival time

## Abstract

**背景与目的:**

表皮生长因子受体酪氨酸激酶抑制剂（epidermal growth factor receptor tyrosine kinase inhibitors, EGFR-TKIs）是含有*EGFR*基因敏感突变肺腺癌患者一线治疗首选药物，但其疗效存在个体差异。已有研究表明核因子E2相关因子2（nuclear factor erythroid-2-related factor 2, Nrf2）在肺癌患者中存在个体表达差异，且其与化疗药物疗效相关。Nrf2激活可抑制EGFR-TKIs在*EGFR*基因突变细胞株中的敏感性。本研究旨在探讨Nrf2在含有*EGFR*基因突变肺腺癌患者中的表达及其与一线EGFR-TKIs疗效的相关性。

**方法:**

应用免疫组化检测31例进展期含有EGFR基因突变的肺腺癌患者组织标本中Nrf2的表达。

**结果:**

含有*EGFR*基因突变的进展期肺腺癌患者中Nrf2表达存在个体差异，Nrf2阳性率为77.4%，Nrf2核高表达率为38.7%；Nrf2在核内的表达水平与EGFR-TKIs缓解程度、无进展生存期（progression free survival, PFS）相关（*P* < 0.05），而与性别、年龄、吸烟、分化程度、*EGFR*基因突变状态和总生存期（Overall survival, OS）无关（*P*>0.05）。Nrf2阳性程度与PFS和OS显著相关（*P* < 0.05），Nrf2阳性组中位PFS、OS低于阴性组（*P* < 0.05）。多因素分析表明Nrf2在肿瘤细胞核内的表达水平是EGFR-TKIs PFS的独立预测因素，Nrf2在肿瘤细胞内的整体阳性程度是OS的独立预测因素（*P* < 0.05）。

**结论:**

Nrf2在含有*EGFR*基因突变的肺腺癌患者中的表达水平与EGFR-TKIs疗效相关，Nrf2可能成为预测EGFR-TKIs疗效的一个理想指标。

## Abstract (en)

**Background and objective:**

Epidermal growth factor receptor tyrosine kinase inhibitors (EGFR-TKIs) have become first-line treatment drugs for lung adenocarcinoma patients with *EGFR* gene mutations. Significant interindividual variations in response rate, progression-free survival (PFS), and overall survival (OS) have been observed. The expression level of nuclear factor erythroid-2-related factor 2 (Nrf2) is related to chemoresistance against platinum drugs. Nrf2 overexpression can inhibit the sensitivity of EGFR-TKIs in cells with EGFR-sensitive mutations. The aim of this study is to investigate the protein expression level of Nrf2 in lung adenocarcinoma patients with *EGFR* gene mutations and to elucidate the correlation between Nrf2 expression and response rate of first-line EGFR-TKIs, as well as PFS and OS.

**Methods:**

Immunohistochemical analysis of Nrf2 in tumor specimens was performed on 31 patients with stage Ⅲ or Ⅳ adenocarcinoma harboring *EGFR* gene mutations.

**Results:**

The Nrf2-positive rate was 77.4%, whereas Nrf2 nuclear high-expression rate was 38.7%. The nuclear expression level of Nrf2 was significantly correlated with response rate (RR) and PFS of EGFR-TKIs (*P* < 0.05), but not with gender, age, smoking, differentiation, and OS (*P*>0.05). The Nrf2-positive level was significantly correlated with PFS and OS of EGFR-TKIs (*P* < 0.05), but not with gender, age, smoking, differentiation, *EGFR* gene mutation status, and RR (*P*>0.05). The PFS and OS of patients with Nrf2-positive expression were significantly shorter than those in patients with negative expression (*P* < 0.05). Furthermore, the nuclear expression level of Nrf2 was the independent predictive factor for EGFR-TKI-induced PFS, and the Nrf2-positive level was the independent predictive factor for EGFR-TKI-induced OS (*P* < 0.05).

**Conclusion:**

The expression level of Nrf2 is significantly correlated with response rate (RR) of EGFR-TKIs, PFS, and OS. Therefore, Nrf2 may be a useful biomarker in predicting response of EGFR-TKIs in patients with advanced-stage lung adenocarcinoma harboring EGFR gene mutations.

肺癌目前是世界范围内发病率和死亡率最高的恶性肿瘤^[1]^，其中80%为非小细胞肺癌（non-small cell lung cancer, NSCLC），约75%的NSCLC就诊时已属于中晚期^[2]^。近年来，多项临床研究表明针对表皮生长因子受体（epidermal growth factor receptor, *EGFR*）基因突变的EGFR酪氨酸激酶抑制剂（EGFR tyrosine kinase inhibitors, EGFR-TKIs）可延长含有*EGFR*基因突变患者的无疾病进展时间（progression free survival, PFS）^[3-7]^，并因其低毒、使用方便越来越为患者所接受。EGFR-TKIs已成为含有*EGFR*基因敏感突变患者一线治疗的首选。*EGFR*突变率在肺腺癌中尤为明显。然而，EGFR-TKIs疗效存在个体差异，因此，探索*EGFR*突变患者中与EGFR-TKIs疗效相关的分子生物指标，对提高药物疗效、提高患者生活质量、减轻患者经济和心理压力显得尤为重要。

Nrf2是细胞调节抗氧化应激和亲电性应激反应的重要转录因子。Nrf2可激活抗氧化反应元件（antioxident response element, ARE）调控的相关耐药基因^[8]^：①细胞内氧化还原基因，如谷氨酸半胱氨酸连接酶、血红素氧合酶-1等；②Ⅱ相解毒基因，如谷胱甘肽-S转移酶、NAD（P）H苯醌氧化还原酶-1（NQO1）等；③编码转运蛋白的基因，如多药耐药蛋白等^[8, 9]^。Nrf2与化疗药物耐药密切相关^[8, 10]^。近期Yamadori等^[11]^发现，在含有*EGFR*基因敏感突变的细胞株中，Nrf2激活可明显降低EGFR-TKIs疗效。但目前尚缺乏Nrf2表达水平与EGFR-TKIs疗效间的相关临床研究。本研究拟通过免疫组织化学方法检测Nrf2表达水平，明确Nrf2表达水平及其与一线EGFR-TKIs疗效及患者预后之间的相关性。

## 1 对象与方法

### 1.1 研究对象

选取2007年1月-2012年5月在北京大学第三医院接受EGFR-TKIs一线治疗的肺腺癌患者。入组标准：按2004年WHO肺癌分类标准病理诊断为肺腺癌者（本研究由于入组患者部分在2011年前入组且病例数较少，未采用2011年肺腺癌国际分类新标准对腺癌进行亚型分类）；含有*EGFR*基因敏感突变；不能手术切除的Ⅲb期和Ⅳ期患者（依据国际肺癌研究协会颁布的第7版分期标准^[12]^）；所有标本为CT引导下肺脏穿刺组织，有足够的组织标本可供免疫组化检测；一线治疗为EGFR-TKIs；预计生存期超过3个月；治疗前、治疗1个月、后每2个-3个月均有影像学检查（胸腹部CT、头颅MRI）。患者治疗前签署知情同意书；所用标本经过北京大学第三医院伦理委员会批准。共纳入符合条件的病例31例，包括男性10例，女性21例；年龄范围24岁-80岁，中位年龄64岁；Ⅲb期患者7例，Ⅳ期患者24例；EGFR外显子19缺失（E19del）突变患者21例，外显子21 L858R（E21L858R）突变10例（31例*EGFR*基因突变检测均为测序法）；接受吉非替尼治疗26例，厄洛替尼3例，埃克替尼2例。

### 1.2 随访及后续治疗

所有患者通过定期来院或电话随访，随访开始时间为2007年1月，末次随访时间为2013年6月1日，最短随访时间3个月，最长60个月。31例患者中后续接受化疗者13例，因脑转移或脊柱转移接受放射治疗者4例。

### 1.3 临床资料收集及疗效评价标准

记录患者临床特征、EGFR TKIs用药记录和影像学指标。疗效指标包括近期和远期疗效。近期疗效按照实体瘤疗效评价标准1.1版^[13]^分为完全缓解（complete response, CR）、部分缓解（partial response, PR）、疾病稳定（stable disease, SD）和疾病进展（progressive disease, PD），获得CR或PR的患者4周或以后确认。远期疗效为PFS和总生存期（overall survival, OS）。PFS定义为从治疗开始至疾病进展或任何原因导致死亡的时间，OS定义为从初次治疗开始至死亡或随访终点时间。

### 1.4 免疫组化检测Nrf2表达

#### 1.4.1 实验方法

活检组织标本经10%甲醛固定后，常规石蜡包埋，4 μm厚度切片，。免疫组化采用SP法（兔抗人Nrf2抗体ab31163）购于Abacm公司，免疫组化二抗SP检测试剂盒购于北京中杉金桥生物技术有限公司，Nrf2抗体按1:100稀释），应用PBS代替一抗作为阴性对照，按照试剂说明书进行操作。

#### 1.4.2 结果判定

采用单盲法阅片（病理医师不清楚临床资料），Nrf2抗原阳性反应可位于细胞浆和细胞核中。随机选取5个中倍镜视野（200倍），每个视野记数200个肿瘤细胞。共计数1, 000个细胞。按染色强度分为：0分：无染色；1分：染色呈淡黄色；2分：染色呈棕黄色；3分：染色呈棕褐色；按核阳性细胞比例（核染色为1分-3分细胞数/计数细胞*100%）分为0-100%。本研究中，参照Solis等^[14]^研究的免疫组化评判标准，将细胞核染色强度和细胞核阳性比例乘积>0定义为Nrf2阳性，反之为阴性。此外将核Nrf2染色强度0分和1分者定义为核Nrf2低表达、2分和3分为核高表达。

### 1.5 统计学方法

应用SPSS 17.0统计学软件分析。率的比较采用卡方检验，或*Fisher*精确检验，相关性检验采用*Pearson*检验，*Kaplan*-*Meier*方法进行生存分析，*Log*-*rank*检验差异性，多因素分析采用*Cox*多因素分析模型。*P* < 0.05为差异有统计学意义。

## 2 结果

### 2.1 随访及疗效

随访率为100%。中位随访时间15个月，随访范围3个月至-60个月。无CR患者，19例（61.3%）PR，7例（22.6%）SD，5例（16.1%）PD。远期疗效：PFS为1个月-50个月，中位PFS 6个月；OS为3个月-60个月，中位OS 15个月。

### 2.2 Nrf2蛋白检测结果及其与临床特征间的关系

Nrf2在细胞核和细胞浆中均可以表达（图 1A-图 1D），存在个体差异。Nrf2核阳性（核染色强度和细胞核阳性细胞比例乘积>0）率为77.4%（24/31），Nrf2阳性水平在性别、年龄、吸烟、分化程度、分期、EGFR基因突变状态以及客观缓解组间无统计学差异（*P*>0.05），见表 1。Nrf2核高表达（细胞核强度>1分者）率为38.7%（12/31），PR患者中核Nrf2高表达率为0（0/19），明显低于SD和PD患者中的100.0%（7/7和5/5）（*P* < 0.001），但核Nrf2表达水平在性别、年龄、吸烟、分化程度、分期、*EGFR*基因突变状态组间无明显差异（*P*>0.05）（表 2）。

**1:**
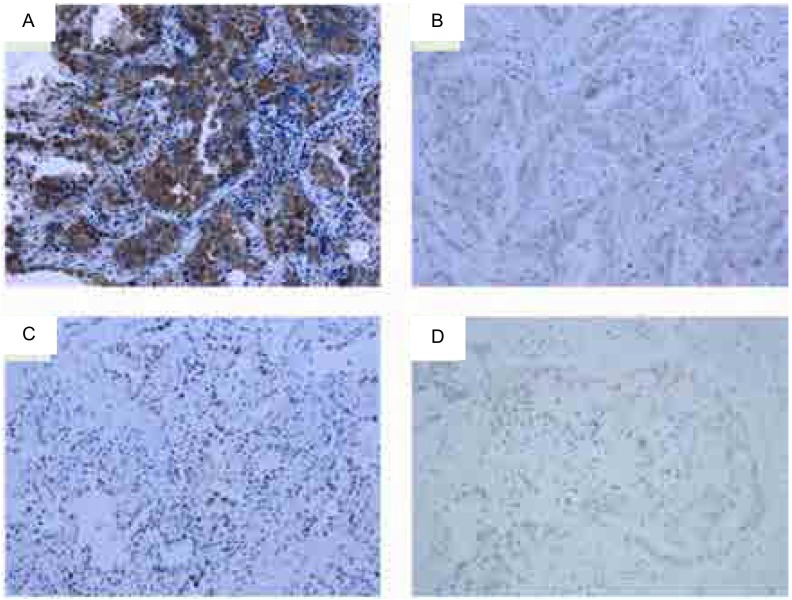
Nrf2在肺腺癌中的表达情况。A：Nrf2胞浆高表达（×200）；B：Nrf2胞浆低表达（×200）；C：Nrf2核高表达（×200）；D：Nrf2核低表达（×200） Expression of Nrf2 in lung adenocarcinoma. A: High expression of Nrf2 in cytoplasm (×200); B: Low expression of Nrf2 in cytoplasm (×200); C: High expression of Nrf2 in nucleus (×200); D: Low expression of Nrf2 in nucleus (×200)

**1:** Nrf2阳性率同肺腺癌患者临床特征间的关系 The relationship between positive Nrf2 and clinical features of lung adenocarcinoma patients

Clinical characterstic	Case (*N*)	Nrf2 positive* rate, *n*[(*n*/*N*)%]	*χ*^2^	*P*
Gender	31		2.562	0.109
Male	10	6 (20.0%)		
Female	21	18 (85.7%)		
Age/(yr)	31		0.524	0.469
< 70	17	14 (82.4%)		
≥70	14	10 (71.4%)		
Smoking history	31		0.215	0.643
Yes	11	8 (72.7%)		
No	20	16 (80.0%)		
Differentiation	31		1.740	0.419
Low	5	5 (100.0%)		
Moderate	15	11 (48.4%)		
High	11	8 (72.7%)		
Staging	31		2.126	0.145
Ⅲ	7	4 (57.1%)		
Ⅳ	24	20 (83.3%)		
*EGFR* mutation	31		1.336	0.248
Exon 19 deletion	21	15 (71.4%)		
Exon 21 L858R	10	9 (90.0%)		
Response	31		2.614	0.271
PR	19	13 (68.4%)		
SD	7	6 (85.7%)		
PD	5	5 (100.0%)		
PR: partial response; SD: stable disease; PD: progressive disease. ^*^Nrf2 Positive refers to score>0, the score was obtained by multiplying the intensity and reactivity extension values of Nrf2 nuclear expression (range 0-300%).				

**2:** 核Nrf2高表达率同肺腺癌患者临床特征间的关系 The relationship between high expression of Nrf2 in nucleus and clinical features of lung adenocarcinoma patients

Clinical characterstic	Case (*N*)	High expression of Nrf2 ^**^ rate, *n*[(*n*/*N*)%]	*χ*^2^	*P*
Gender	31		0.472	0.492
Male	10	3 (30.0%)		
Female	21	9(42.9%)		
Age (yr)	31		1.106	0.293
< 70	17	8(47.1%)		
≥70	14	4 (28.6%)		
Smoking history	31		0.040	0.842
Yes	11	4(36.4%)		
No	20	8 (40.0%)		
Differentiation	31		1.164	0.559
Low	5	3 (60.0%)		
Moderate	15	5 (33.3%)		
High	11	4 (36.4%)		
Staging	31		0.392	0.531
Ⅲ	7	2 (28.6%)		
Ⅳ	24	10 (41.7%)		
*EGFR* mutation	31		0.010	0.919
Exon-19 deletion	21	8 (38.1%)		
Exon-21 L858R	10	4 (40.0%)		
Response	31		31.000	< 0.001
PR	19	0 (0)		
SD	7	7 (100.0%)		
PD	5	5(100.0%)		
^**^High expression of Nrf2 refer to Nrf2 nuclear high intensity, score 2 or 3.				

### 2.3 Nrf2表达水平与EGFR-TKIs缓解程度、PFS、OS相关性分析

Nrf2阳性水平与EGFR-TKIs缓解程度（*r*=0.290, *P*=0.113）无关，但与PFS（*r*=-0.590, *P* < 0.001）和OS（*r*=-0.694, *P* < 0.001）相关，Nrf2阳性者的PFS及OS低于Nrf2阴性者。Nrf2核高表达水平与EGFR-TKI缓解程度（*r*=-0.914, *P* < 0.001）和PFS（*r*=-0.485, *P*=0.006）相关，但与OS（*r*=-0.227, *P*=0.219）无关。Nrf2核高表达者的缓解程度和PFS低于Nrf2核低或不表达者。

### 2.4 生存分析

*Kaplan*-*Meier*生存分析表明：①Nrf2阳性组PFS和OS低于阴性组。其中位PFS分别为6个月和12个月（*P*=0.057）（图 2A）；中位OS分别为15个月和54个月（*P*=0.013）（图 2B）。②核内Nrf2高表达组PFS明显低于低或不表达组，二者OS无明显差异。其中位PFS分别4个月和10个月（*P* < 0.001）（图 2C）；中位OS分别为12个月和30个月（*P*=0.331）（图 2D）。③患者PFS和OS与EGFR突变状态无关。EGFR E19Del突变和E21 L858R突变的中位PFS分别9个月和5个月（*P*=0.890）（图 2E）；中位OS分别为17个月和15个月（*P*=0.848）（图 2F）。

**2:**
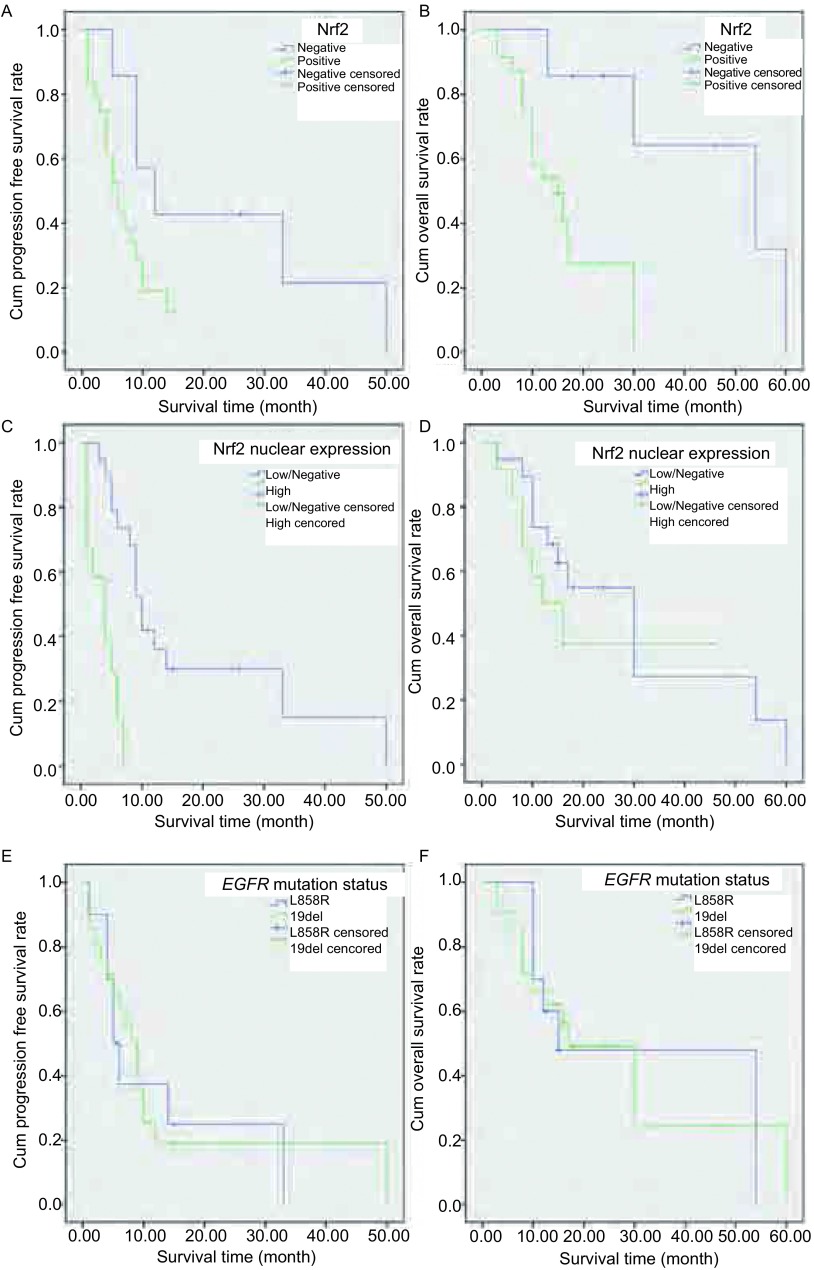
*Kaplan*-*Meier*累计生存时间曲线分析。A：Nrf2阳性组和阴性组患者的PFS；B：Nrf2阳性组和阴性组患者的OS；C：Nrf2核高表达组和低或不表达组患者的PFS；D：Nrf2核高表达组和低或不表达组患者的OS；E：E19del突变和E21L858R突变组患者的PFS；F：E19del突变和E21L858R突变组患者的OS *Kaplan*-*Meier* cumulative survival time curves analysis. A: PFS between Nrf2 positive expression and negative expression in groups of patients with lung adenocarcinoma; B: OS between Nrf2 positive expression and negative expression in groups of patients with lung adenocarcinoma; C: PFS between Nrf2 nuclear high expression and negative/low expression in groups of patients with lung adenocarcinoma; D: OS between Nrf2 nuclear high expression and negative/low expression in groups of patients with lung adenocarcinoma; E: PFS between E19del and E21L858R in groups of patients with lung adenocarcinoma patients; F: OS between E19del and E21L858R in groups of patients with lung adenocarcinoma patients

### 2.5 *Cox*多因素回归分析

在校对患者*EGFR*基因突变状态、分期、Nrf2核内表达、Nrf2阳性等因素后，多因素分析表明核Nrf2表达水平是EGFR-TKIs PFS的独立预测因素（*P*=0.002）；Nrf2阳性水平是OS的独立预测因素（*P*=0.042），见表 3。

**3:** 多因素分析EGFR-TKIs特异性生存的预后因素(*n*=31) Cox regression analysis of the disease-specific survival with EGFR-TKIs (*n*=31)

Characteristic	Regression coefficient *β*	Standard error	Wald	*P*	Exp (B)	95%CI
Progression-free survival						
Stage	0.491	0.567	0.749	0.387	1.634	0.538-4.694
EGFR mutation	0.200	0.451	0.197	0.658	1.221	0.505-2.953
Nrf2 score	0.726	0.591	1.511	0.219	2.067	0.649-6.583
Nrf2 nuclear expression	1.782	0.579	9.483	0.002	5.944	1.912-18.483
Overall survival (OS)						
Stage	0.246	0.658	0.139	0.709	1.279	0.352-4.647
*EGFR* mutation	0.038	0.528	0.139	0.709	1.039	0.369-2.925
Nrf2 score	1.695	0.833	4.145	0.042	5.449	1.065-27.873
Nrf2 nuclear expression	0.301	0.521	0.335	0.563	1.352	0.487-3.752

## 3 讨论

*EGFR*基因敏感突变的肺癌患者一线首选EGFR-TKIs已成为治疗共识。*EGFR*基因突变集中在*EGFR*基因的18-21外显子，其中E19Del和E21 L858R占90%以上，二者也是EGFR TKIs最常见的敏感突变^[15, 16]^。但仍有20%-40%的患者服药无效^[4-7]^，且有效患者获益程度个体差异明显，不论是应用第一代TKIs中的吉非替尼、厄洛替尼、埃克替尼，还是第二代的阿法替尼都存在这种情况。因此，寻找预测EGFR-TKIs疗效的指标成为了研究热点。虽然针对*EGFR*突变本身、EGFR通路相关分子以及其他通路分子的研究已部分证实和EGFR TKIs耐药有关，如T790M突变、*EGFR*基因突变丰度、*PTEN*、*PI3K*以及*c*-*MET*基因扩增等^[16-19]^，但尚不足解释EGFR-TKIs在*EGFR*基因突变患者中的疗效差异。

Nrf2是调控细胞应对氧化应激和亲电性应激反应的重要转录因子。Nrf2通过调控ARE驱动的药物代谢酶和抗氧化酶/蛋白的表达，来保护细胞免受或减少外来有毒物质的杀伤^[9]^。Nrf2的这种保护作用与肿瘤耐药密切相关。多项研究表明Nrf2过表达与肺癌细胞耐药有关。但缺乏与EGFR-TKIs疗效相关的临床研究。

本研究通过免疫组化方法检测31例含有*EGFR*基因敏感突变的肺腺癌患者，结果发现Nrf2阳性率达77.4%，远高于Solis等^[14]^报道的阳性率26.0%，且也高于曹宝山等^[20]^前期研究中的报道34.0%。产生差异的原因在于：①前期小样本研究发现晚期患者Nrf2阳性表达远高于早期患者^[20]^，而Solis等^[14]^研究以早期患者为主，晚期患者不足1/4，或许是产生差异原因之一；②Yamadori等^[11]^研究发现EGFR信号活化可上调Nrf2表达水平，因此在EGFR敏感基因突变患者中，EGFR通路活化是导致Nrf2阳性表达率增高原因之一。但是在Solis等^[14]^报道的23例*EGFR*基因突变患者中无Nrf2阳性表达，此种差异或许与肿瘤分期、种族等因素有关；③本研究为回顾性研究，样本量较小，选择偏倚不能除外。因此，可进行前瞻性大样本研究证实。本研究中未观察到Nrf2表达水平与吸烟、性别有关，与Solis等^[14]^报道不同，或许与EGFR通路激活，抵消了吸烟等刺激导致的区别以及本研究入组病例数偏少有关。

本研究发现核Nrf2高表达与EGFR TKIs缓解程度相关，高表达组中无1例患者获得PR。这与Yamadori等^[11]^在含有*EGFR*基因敏感突变的细胞株中发现的现象一致，即Nrf2激活可明显降低EGFR-TKIs疗效^[11]^。且Nrf2在细胞核内高表达对EGFR-TKIs缓解程度的预测能力明显高于Nrf2阳性（细胞核染色强度和细胞阳性比例的乘积）的能力。这或许是因为：Nrf2正常情况在胞浆中表达，一旦进入细胞核中，其可激活ARE调控的药物解毒酶和代谢酶，并促进细胞增殖、抑制细胞凋亡^[8, 9]^。本研究生存分析表明：核Nrf2表达水平、Nrf2阳性水平与患者的PFS均相关，且Nrf2阳性水平与患者生存期呈负相关。这与Solis^[14]^和Yang等^[21]^研究结果相一致。提示Nrf2表达既可能是EGFR-TKIs疗效的预测指标，也是预后的预测指标。但在本研究中，发现EGFR不同突变位点患者中其PFS和OS无差异，这与Jackman等^[22]^研究结果不同，其发现外显子19缺失突变患者生存期优于外显子21突变。这或许与本研究样本量小有关。

目前已经清楚，Nrf2可通过外来刺激、Nrf2突变、Keap1突变或Keap1甲基化获得激活^[10, 23, 24]^。具体何种机制在本研究患者群中发挥作用有待深入研究。

本研究的主要限制在于样本量偏小，存在选择偏倚可能。但本研究发现Nrf2表达水平与EGFR-TKI近期和远期疗效相关，Nrf2核表达水平是PFS的独立预测因子，Nrf2阳性水平是OS的独立预测因子，因此Nrf2在含有*EGFR*突变患者中，可能成为预测EGFR-TKIs疗效的的理想指标。我们将进一步扩大样本量进行前瞻性研究，以验证Nrf2对预测EGFR-TKI疗效和预后的临床价值。

## References

[1] Jemal A, Bray F, Center MM (2011). Global cancer statistics. CA Cancer J Clin.

[2] Mountain CF (1997). Revisions in the International System for Staging Lung Cancer. Chest.

[3] Ku GY, Haaland BA, de Lima Lopes G (2011). Gefitinib *vs*. chemotherapy as first-line therapy in advanced non-small cell lung cancer: *meta*-analysis of phase Ⅲ trials. Lung Cancer.

[4] Mitsudomi T, Morita S, Yatabe Y (2010). Gefitinib versus cisplatin plus docetaxel in patients with non-small-cell lung cancer harbouring mutations of the epidermal growth factor receptor (WJTOG3405): an open label, randomised phase 3 trial. Lancet Oncol.

[5] Mok TS, Wu YL, Thongprasert S (2009). Gefitinib or carboplatin-paclitaxel in pulmonary adenocarcinoma. N Engl J Med.

[6] Rosell R, Carcereny E, Gervais R (2012). Erlotinib versus standard chemotherapy as first-line treatment for European patients with advanced *EGFR* mutation-positive non-small-cell lung cancer (EURTAC): a multicentre, open-label, randomised phase 3 trial. Lancet Oncol.

[7] Zhou C, Wu YL, Chen G (2011). Erlotinib versus chemotherapy as first-line treatment for patients with advanced *EGFR* mutation-positive non-small-cell lung cancer (OPTIMAL, CTONG-0802): a multicentre, open-label, randomised, phase 3 study. Lancet Oncol.

[8] Que LL, Wang HX, Cao BS (2011). The regulation and functions of transcription factor Nrf2 in cancer chemoprevention and chemoresistance. J Chin Pharm Sci.

[9] Hayes JD, McMahon M, Chowdhry S (2010). Cancer chemoprevention mechanisms mediated through the Keap1-Nrf2 pathway. Antioxid Redox Signal.

[10] Singh A, Misra V, Thimmulappa RK (2006). Dysfunctional KEAP1-NRF2 interaction in non-small-cell lung cancer. PLoS Med.

[11] Yamadori T, Ishii Y, Homma S (2012). Molecular mechanisms for the regulation of Nrf2-mediated cell proliferation in non-small-cell lung cancers. Oncogene.

[12] Detterbeck FC, Boffa DJ, Tanoue LT (2009). The new lung cancer staging system. Chest.

[13] Eisenhauer EA, Therasse P, Bogaerts J (2009). New response evaluation criteria in solid tumours: revised RECIST guideline (version 1.1). Eur J Cancer.

[14] Solis LM, Behrens C, Dong W (2010). Nrf2 and Keap1 abnormalities in non-small cell lung carcinoma and association with clinicopathologic features. Clin Cancer Res.

[15] Sharma SV, Bell DW, Settleman J (2007). Epidermal growth factor receptor mutations in lung cancer. Nat Rev Cancer.

[16] Chen ZY, Zhong WZ, Zhang XC (2012). EGFR mutation heterogeneity and the mixed response to EGFR tyrosine kinase inhibitors of lung adenocarcinomas. Oncologist.

[17] Su KY, Chen HY, Li KC (2012). Pretreatment epidermal growth factor receptor (EGFR) T790M mutation predicts shorter EGFR tyrosine kinase inhibitor response duration in patients with non-small-cell lung cancer. J Clin Oncol.

[18] Soria JC, Mok TS, Cappuzzo F (2012). *EGFR*-mutated oncogene-addicted non-small cell lung cancer: current trends and future prospects. Cancer Treat Rev.

[19] Zhou Q, Zhang XC, Chen ZH (2011). Relative abundance of *EGFR* mutations predicts benefit from gefitinib treatment for advanced non-small-cell lung cancer. J Clin Oncol.

[20] Cao BS, Zhu X, Yin WC (2012). The role of the expression level of Nrf2 in predicting chemoresistance and prognosis in advanced non-small cell lung cancer. TUMOR.

[21] Yang H, Wang W, Zhang Y (2011). The role of NF-E2-related factor 2 in predicting chemoresistance and prognosis in advanced non-small-cell lung cancer. Clin Lung Cancer.

[22] Jackman DM, Yeap BY, Sequist LV (2006). Exon 19 deletion mutations of epidermal growth factor receptor are associated with prolonged survival in non-small cell lung cancer patients treated with gefitinib or erlotinib. Clin Cancer Res.

[23] Yoo NJ, Kim HR, Kim YR (2012). Somatic mutations of the *KEAP1* gene in common solid cancers. Histopathology.

[24] Muscarella LA, Parrella P, D'Alessandro V (2011). Frequent epigenetics inactivation of *KEAP1* gene in non-small cell lung cancer. Epigenetics.

